# Lightweight deep learning framework for intracranial hemorrhage detection in brain CT scans

**DOI:** 10.3389/fmed.2026.1804084

**Published:** 2026-06-17

**Authors:** Sumaira Hussain, Salman Jan, Manal Aldhayan, Mohammad Asmat Ullah Khan, Shahid Kamal, Abid Jameel, Jawad Hasan Alkhateeb, Jamal Zraqou

**Affiliations:** 1 School of Computer Science and Technology, Shandong Jianzhu University, Jinan, China; 2 Faculty of Computer Studies, Arab Open University-Bahrain, A’ali, Bahrain; 3 Center for Advanced Analytics, CoE for Artificial Intelligence, Faculty of Computing and Informatics, Multimedia University, Cyberjaya, Selangor, Malaysia; 4 Department of Computer Science, College of Computer and IT, Shaqra University, Shaqra, Saudi Arabia; 5 Department of Computer Science, College of Computer and Information Sciences, Prince Sultan University, Riyad, Saudi Arabia; 6 Department of Computer Science, Faculty of Computing, International Islamic University Islamabad, Islamabad, Pakistan; 7 Computer Engineering Department, College of Engineering and Computer Science, Prince Mohammad Bin Fahd University, Khobar, Saudi Arabia; 8 Department of Computer Science, University of Petra, Amman, Jordan

**Keywords:** automated diagnosis, brain CT, deep learning, EfficientNet-B0, intracranial hemorrhage, lightweight CNN, MobileNetV2, transfer learning

## Abstract

Intracranial hemorrhage, including clinically significant intracranial hemorrhage conditions, is a life-threatening condition in which rapid and accurate detection through brain computed tomography (CT) scans is crucial for patient survival. Manual interpretation of these scans remains time-consuming and may vary between observers, leading to potential diagnostic delays. This study investigates the application of lightweight and mobile-optimized deep-learning models for the automated detection of intracranial hemorrhage using a curated subset of a publicly available brain CT hemorrhage dataset, for automated intracranial hemorrhage detection. A custom convolutional neural network (CNN) and two transfer-learning architectures MobileNetV2 and EfficientNet-B0 were evaluated in terms of diagnostic accuracy, generalization, and computational efficiency. Among the tested models, MobileNetV2 demonstrated the highest overall performance, achieving an accuracy of 87% and an AUC of 0.94, while the lightweight CNN achieved 79% accuracy. EfficientNet-B0 also showed competitive results but required greater computational resources. The findings demonstrate that lightweight neural architectures can achieve reliable diagnostic performance while remaining suitable for assistive decision-support applications, although further improvement in sensitivity and clinical validation are required. The study highlights that carefully optimized deep-learning systems can support preliminary clinical assessment; however, additional validation and performance refinement are necessary before practical real-world deployment.

## 1 Introduction

Intracranial hemorrhage (ICH) is a critical neurological condition that requires rapid diagnosis and clinical intervention to reduce mortality and long-term neurological complications. Computed tomography (CT) is the primary imaging modality used for detecting hemorrhagic abnormalities because of its speed, accessibility, and effectiveness in emergency settings. However, manual interpretation of CT scans remains time-consuming and subject to observer variability, particularly in subtle or diffuse hemorrhagic cases. These challenges have motivated increasing interest in artificial-intelligence (AI)-based systems for automated hemorrhage detection and assistive clinical analysis.

Deep learning (DL) techniques, especially convolutional neural networks (CNNs), have shown outstanding success in medical-image classification and segmentation (1, 2). They can automatically learn hierarchical visual features without handcrafted descriptors, enabling effective detection of subtle pathological patterns. Recent studies have demonstrated DL’s utility in intracranial hemorrhage classification (3, 4), stroke detection (5), and brain-tumor recognition (6). Despite these achievements, many high-performing models, such as VGG-19 or ResNet-152, demand extensive computational resources and are impractical for deployment in emergency or mobile diagnostic settings (7).

Lightweight and mobile-optimized architectures, including MobileNet and EfficientNet families, provide an appealing alternative by balancing accuracy with computational efficiency (8, 9). These models employ depthwise-separable and compound-scaled convolutions that drastically reduce parameter count while maintaining strong representational power. Integrating such models into hospital information systems or portable CT-scanner interfaces has the potential to assist radiologists in preliminary decision-making, subject to further validation, improved sensitivity, and comprehensive clinical evaluation.

This study proposes and evaluates a lightweight deep-learning framework for automated intracranial hemorrhage detection using a curated subset of a publicly available brain CT hemorrhage dataset. Three architectures were compared: a custom lightweight CNN, MobileNetV2, and EfficientNet-B0. The objective was to assess how much diagnostic performance can be retained when moving from heavier CNNs toward lighter models suitable for clinical deployment. Comparative experiments were conducted under identical conditions to ensure fair evaluation, reporting classification accuracy, precision, recall, F1-score, and area under the ROC curve (AUC).

Unlike prior studies that primarily focus on maximizing classification accuracy using computationally intensive architectures or proprietary datasets, this study emphasizes a reproducible and efficiency-oriented evaluation of lightweight deep-learning models for intracranial hemorrhage detection. The main contribution lies in the systematic comparison of lightweight and transfer-learning architectures under standardized conditions using a publicly available CT dataset, with a particular focus on balancing diagnostic performance and computational efficiency. This work aims to provide practical insights for developing deployable and resource-efficient AI-assisted diagnostic systems.

The rest of the paper is organized as follows: section 2 reviews related work on deep learning for hemorrhage detection; section 3 explains the dataset and methods; section 4 presents experimental results and performance comparison; and section 5 concludes with key findings and future directions.

## 2 Related work

The automated interpretation of brain-imaging data for hemorrhage detection has progressed remarkably in the past decade. Early computer-aided diagnosis (CAD) systems relied on classical machine-learning (ML) techniques such as support-vector machines (SVMs), random forests, or k-nearest-neighbor classifiers, which required handcrafted features derived from pixel intensities, gray-level co-occurrence matrices, or wavelet decompositions (10). Although these methods provided foundational insights, their performance was highly dependent on expert-driven feature selection and limited generalization across scanners or institutions (11). In heterogeneous clinical datasets, even subtle differences in slice thickness or contrast levels could drastically reduce accuracy, underlining the need for data-driven feature learning. However, these approaches often lack scalability and robustness when applied to heterogeneous clinical datasets, limiting their practical applicability.

The emergence of deep learning (DL) revolutionized medical-image analysis by allowing hierarchical feature extraction directly from raw data. Convolutional neural networks (CNNs) rapidly became the dominant architecture for hemorrhage detection because of their ability to learn spatial and contextual patterns without manual intervention (12). Patel et al. (1) implemented a CNN trained on non-contrast CT scans to detect intracranial hemorrhage, achieving significant improvements over handcrafted-feature baselines. Building on this, Chilamkurthy et al. (2) introduced a multitask CNN capable of identifying five hemorrhage subtypes subarachnoid, intraventricular, intraparenchymal, subdural, and epidural demonstrating the feasibility of automated triage systems for emergency departments. Despite these promising results, many of these models rely on deep and computationally intensive architectures, which limit their feasibility for real-time or resource-constrained clinical environments. Other studies leveraged residual networks (ResNet) or densely connected networks (DenseNet) to refine classification accuracy and localization precision (3, 4).

Despite these advances, high-capacity models such as ResNet-152 or VGG-19 remain computationally expensive, often exceeding hundreds of millions of parameters. Their large memory footprint and inference latency hinder real-time clinical deployment, especially in mobile or low-resource environments (5). This challenge has motivated a shift toward lightweight and transfer-learning frameworks, which reuse pretrained feature extractors and optimize for speed without compromising diagnostic reliability. The introduction of MobileNet, ShuffleNet, and EfficientNet families marked an important turning point, employing depthwise-separable and compound-scaled convolutions to drastically reduce computation while preserving expressive power (6, 7).

However, existing studies often focus on individual model performance without providing a systematic and reproducible comparison of lightweight architectures under standardized experimental conditions, leaving a gap in understanding the trade-offs between diagnostic accuracy and computational efficiency.

Several researchers have validated these lightweight architectures for intracranial-hemorrhage tasks. Iqbal et al. (8) applied MobileNetV2 to CT slices and achieved accuracy comparable to ResNet-50 while using only a fraction of its parameters. Phong et al. (9) explored EfficientNet-B0, reporting improved stability and generalization across multiple datasets through compound scaling of depth, width, and resolution. Homayoun et al. (13) combined MobileNet features with recurrent layers to capture temporal dependencies between contiguous CT slices, further enhancing classification robustness. These studies collectively indicate that lightweight models can retain diagnostic performance while substantially reducing computational cost.

In parallel, attention mechanisms and interpretability tools have been introduced to strengthen clinical trust in DL outputs. Hong et al. (14) incorporated Grad-CAM heat-map visualization to highlight hemorrhagic regions on CT scans, helping radiologists verify AI-based predictions. Liu et al. (15) integrated vision transformers (ViTs) with CNN backbones, illustrating that hybrid approaches can improve sensitivity to subtle SAH regions. Moreover, large-scale reviews by Kermany et al. (16) and Wiśniewski et al. (17) emphasize that transparent, computationally efficient, and reproducible DL frameworks are essential for widespread diagnostic adoption. Table 1 summarizes the related work.

**TABLE 1:** Summary of related work in hemorrhage detection and lightweight deep-learning models.

Study/dataset	Methodology	Target task	Key findings	Limitations
Early ML (SVM, KNN, RF) (10, 11)	Handcrafted texture and intensity features + classical ML classifiers	Intracranial hemorrhage (ICH) classification	Provided foundational results for automatic detection using small datasets	Poor generalization; dependent on manual feature engineering
Rayan et al. (12)	CNN trained on non-contrast CT images	Hemorrhage detection and localization	Demonstrated strong improvement over traditional ML methods	Limited dataset; lacked multi-class capability
Chilamkurthy et al. (1)	Multi-task CNN framework	Detection of five ICH subtypes including SAH	Achieved radiologist-level sensitivity; validated on CQ500 dataset	Computationally heavy; unsuitable for real-time use
Wang et al. (2)	Hybrid CNN–RNN sequence modeling	Hemorrhage subtype classification (incl. SAH)	Captured temporal correlation between slices; improved recall	Model complexity; long training time
RSNA ICH Dataset (> 25 000 CT scans) (3)	Large-scale CNN competition models	Multi-class hemorrhage detection	Reported AUC > 0.95 across subtypes	Requires high compute resources; not reproducible on small setups
CQ500 dataset (4)	Expert-annotated CT benchmark	ICH and SAH detection	Provided high-quality public benchmark labels	Limited size (∼500 cases); narrow diversity
Public brain CT hemorrhage dataset (curated subset used in this study)	Publicly available CT dataset curated for hemorrhage vs. non-hemorrhage classification	Hemorrhage vs. Non-hemorrhage classification	Suitable for reproducible prototyping and benchmarking	Small dataset; requires augmentation for balance
Sandler et al. (MobileNetV2) (6)	Depthwise-separable CNN architecture	Generic medical-image tasks	Achieved competitive accuracy with > 10 × fewer parameters	Limited use in neuro-CT applications
Tan and Le (EfficientNet-B0) (7)	Compound-scaled CNN architecture	Transfer learning for medical imaging	Balanced accuracy and efficiency through scaling	Requires fine-tuning for small datasets
Iqbal et al. (8)	MobileNetV2 transfer learning on CT	Intracranial hemorrhage subtype classification	Comparable to ResNet-50 with lower computation	Dataset-specific; lacks generalization study
Phong et al. (2022) (9)	EfficientNet-B0 fine-tuned model	Head-CT hemorrhage classification	Reported AUC ≈ 0.93; strong generalization	Needs external validation
Homayoun et al. (13)	MobileNet + Recurrent Layer Fusion	Sequential CT slice analysis	Improved temporal stability and F1-score	Complex integration; higher inference latency
Hong et al. (14)	Grad-CAM visual explanations for CNNs	Hemorrhage region localization on CT	Enhanced clinician trust via heat-map interpretation	Adds explainability but not core accuracy
Liu et al. (15)	CNN–vision transformer hybrid	SAH region detection and classification	Improved sensitivity to small lesions	Computationally intensive; early stage research
Kermany et al. (16) and Wiśniewski et al. (17)	Survey of DL in medical imaging	General AI diagnostic frameworks	Highlighted need for reproducibility and lightweight AI models	Reviews; no empirical evaluation

Despite notable progress in applying deep learning for intracranial hemorrhage detection, most existing studies rely on computationally intensive architectures or proprietary datasets, which limit reproducibility and practical deployment in real-world clinical settings. Furthermore, there is limited emphasis on systematically evaluating lightweight architectures under consistent experimental conditions, particularly in understanding the trade-offs between diagnostic performance and computational efficiency.

To address these gaps, this study provides a reproducible and efficiency-oriented comparative analysis of lightweight and transfer-learning models for intracranial hemorrhage detection using a curated CT dataset. The proposed framework aims to balance diagnostic accuracy with computational efficiency, offering practical insights for the development of deployable and resource-efficient AI-assisted diagnostic systems.

## 3 Materials and methods

The proposed deep-learning framework is developed to enable automated detection and classification of intracranial hemorrhage from brain CT scans. The methodology comprises data acquisition, preprocessing, model development, training, and evaluation stages designed to ensure both diagnostic accuracy and computational efficiency.

### 3.1 Dataset description

**FIGURE 1:**
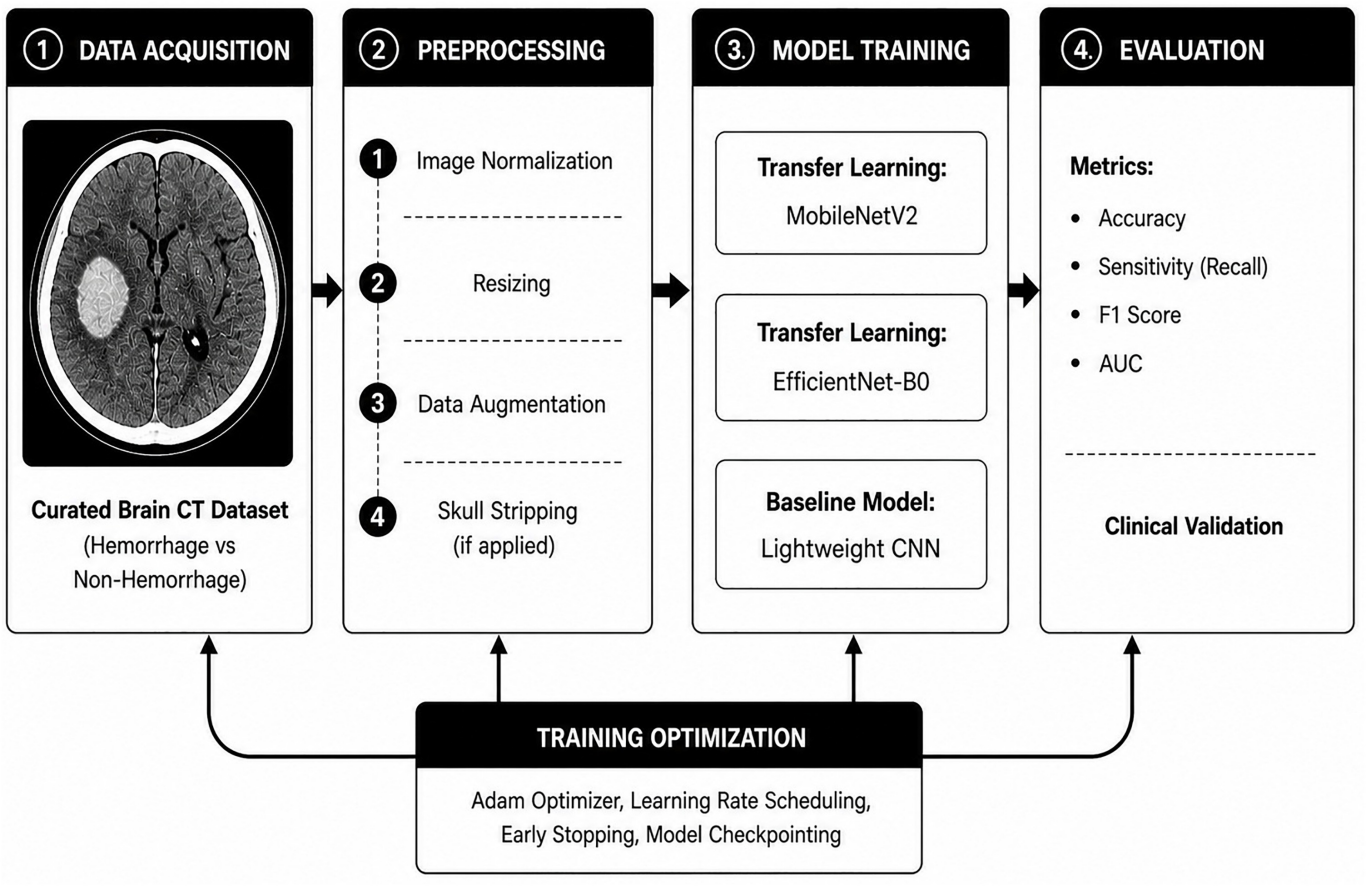
Overall workflow of the proposed lightweight deep-learning framework for intracranial hemorrhage detection.

This study utilized a curated subset of the publicly available Kaggle Brain CT Hemorrhage dataset (5), which contains axial brain CT slices categorized as hemorrhagic and non-hemorrhagic cases. A total of 3,180 CT images were selected for this study, including 1,590 hemorrhagic and 1,590 non-hemorrhagic images to maintain balanced class representation during training and evaluation. The subset was prepared to ensure balanced representation of both classes and consistent experimental evaluation across all models. Figure 1 block diagram showing the overall workflow of the proposed research.

The dataset includes axial slices from anonymized patients, ensuring compliance with data privacy and ethical standards. No patient-identifiable information or clinical metadata are included, allowing unrestricted academic use without institutional review board (IRB) approval.

**FIGURE 2:**
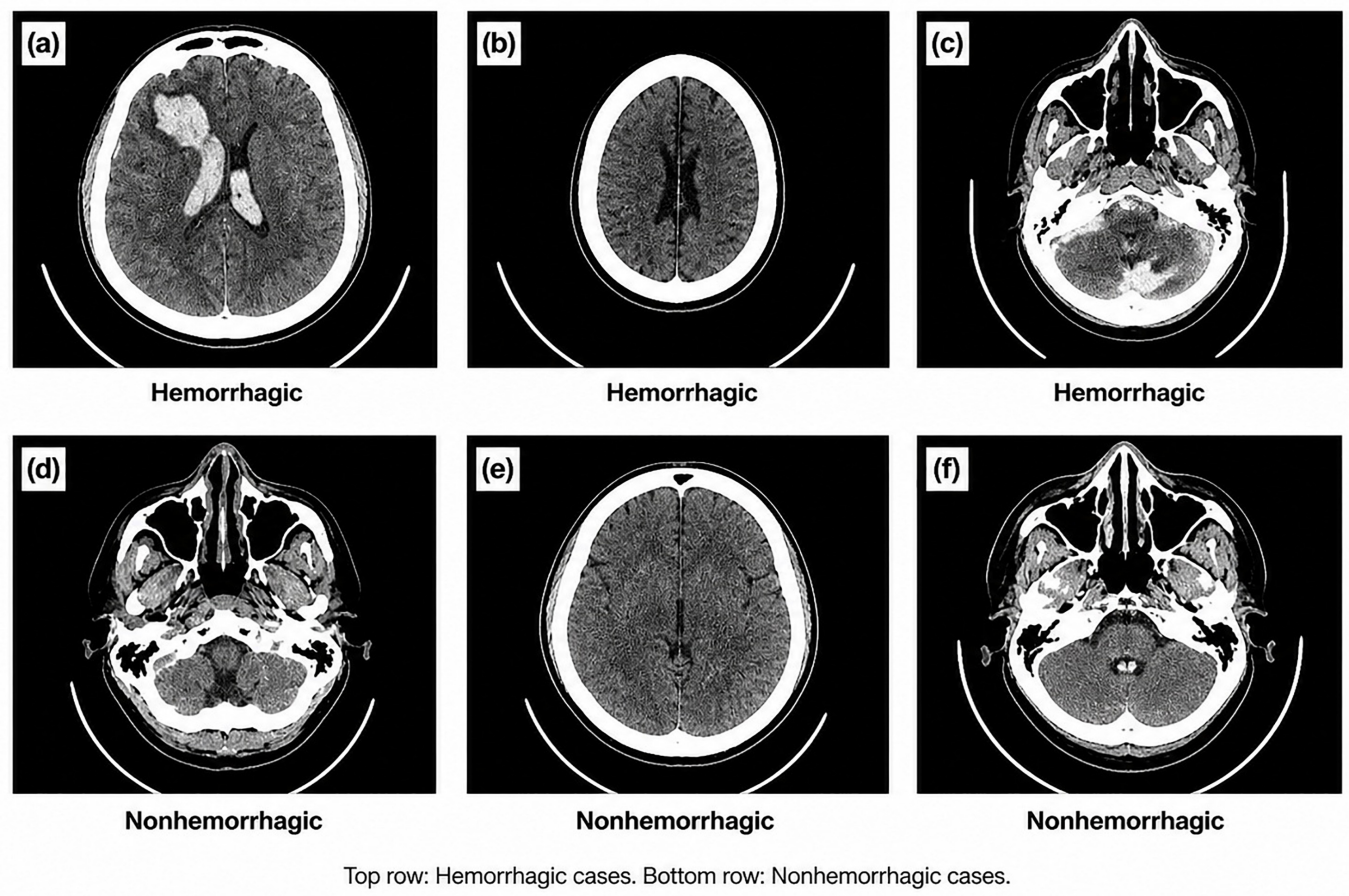
Representative samples of hemorrhagic **(a–c)** and non-hemorrhagic **(d–f)** brain CT images from the curated dataset used in this study. Images are shown from different anatomical slices to illustrate variability in appearance.

Figure 2 illustrates representative examples of input images for both classes Hemorrhagic and (Normal) Cases. The dataset contained an approximately balanced class distribution, ensuring fair representation of hemorrhagic and non-hemorrhagic cases. Images were resized to 224 × 224 pixels to match input dimensions of standard CNN architectures. The final curated subset consisted of 1,590 hemorrhagic and 1,590 non-hemorrhagic CT images. Based on the 70:15:15 stratified split, the testing subset contained approximately 477 images while preserving class balance across all subsets.

The dataset was randomly divided into three subsets following a 70:15:15 ratio for training, validation, and testing, respectively. The dataset was divided using a stratified slice-level train-validation-test split (70:15:15), as patient-level identifiers were not publicly available in the dataset. While stratification preserved class balance across subsets, strict patient-level separation could not be independently verified, which may introduce potential data leakage and optimistic performance estimates. This limitation is acknowledged in the interpretation of the results.

### 3.2 Data preprocessing and augmentation

To enhance model robustness and mitigate overfitting, several preprocessing steps were applied. All images were resized to 224 × 224 pixels and converted to RGB format to ensure compatibility with pretrained architectures. Pixel intensities were normalized to the range [-1, 1] using mean–variance normalization.

Data augmentation techniques were applied during training, including random horizontal flipping, rotation within a range of ± 15 degrees, and minor brightness and contrast adjustments. These transformations were applied probabilistically to increase data diversity and improve model generalization.

All preprocessing and augmentation operations were implemented using the PyTorch torchvision library, ensuring consistency across all experiments.

During inference, binary classification decisions were generated using a sigmoid activation threshold of 0.5. All preprocessing operations were applied consistently across training, validation, and testing subsets to ensure experimental reproducibility.

### 3.3 Model architecture

Three models were evaluated to examine the relationship between architectural complexity and diagnostic performance:

#### 3.3.1 Lightweight CNN (baseline)

A custom-designed CNN composed of three convolutional blocks, each followed by batch normalization, ReLU activation, and max-pooling layers. Depthwise separable convolutions were employed to reduce parameters, and a global average pooling layer preceded a sigmoid classifier for binary output.

**FIGURE 3:**
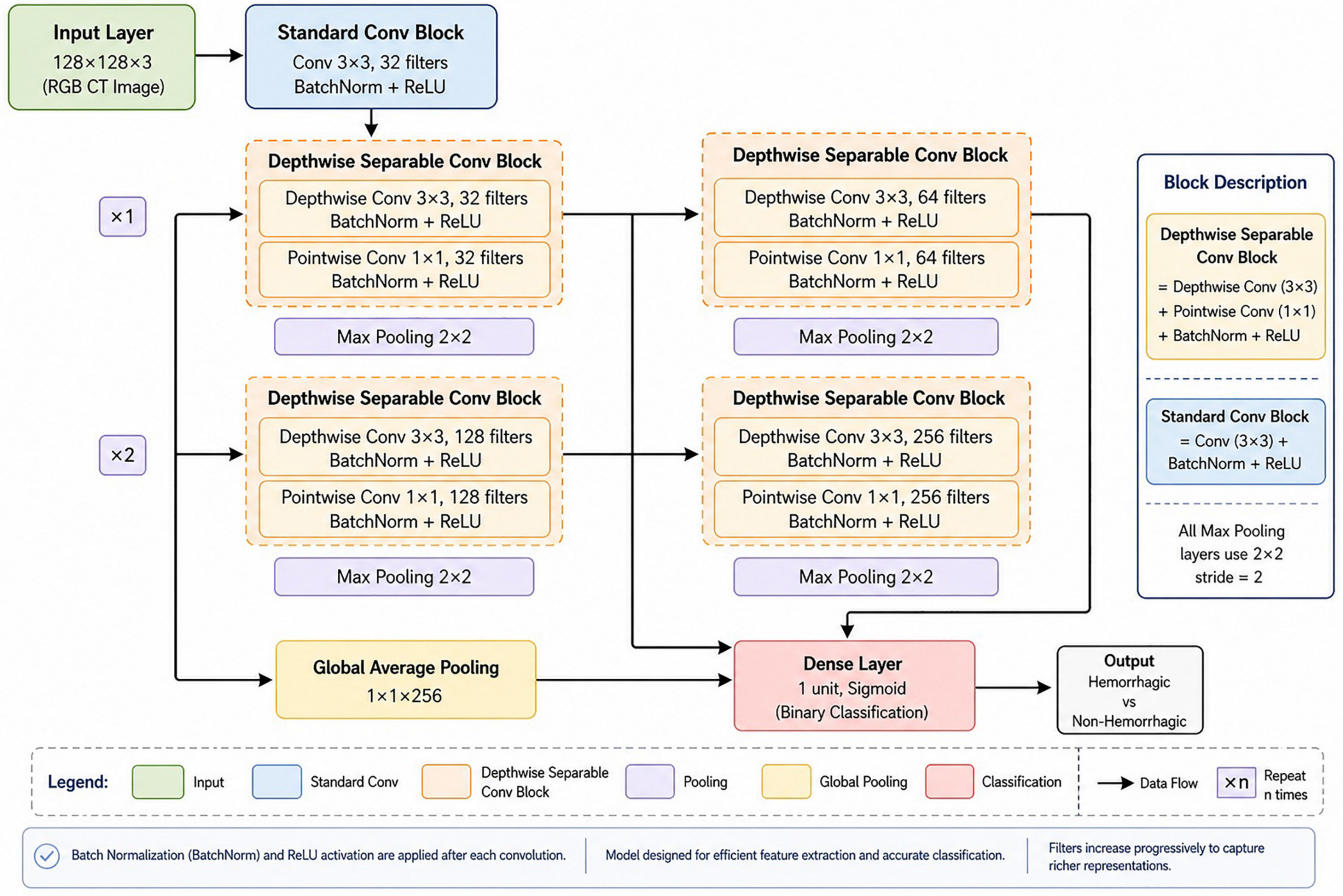
Architecture of the proposed lightweight convolutional neural network (CNN) model. The network consists of standard convolutional and depth wise separable convolution blocks, followed by global average pooling and a dense classification layer. Skip connections are incorporated to enhance feature propagation and model efficiency.

The total parameter count was approximately 1.2 million, making it computationally efficient for real-time inference. The architecture of the proposed lightweight CNN model is illustrated in Figure 3.

#### 3.3.2 MobileNetV2

**FIGURE 4:**
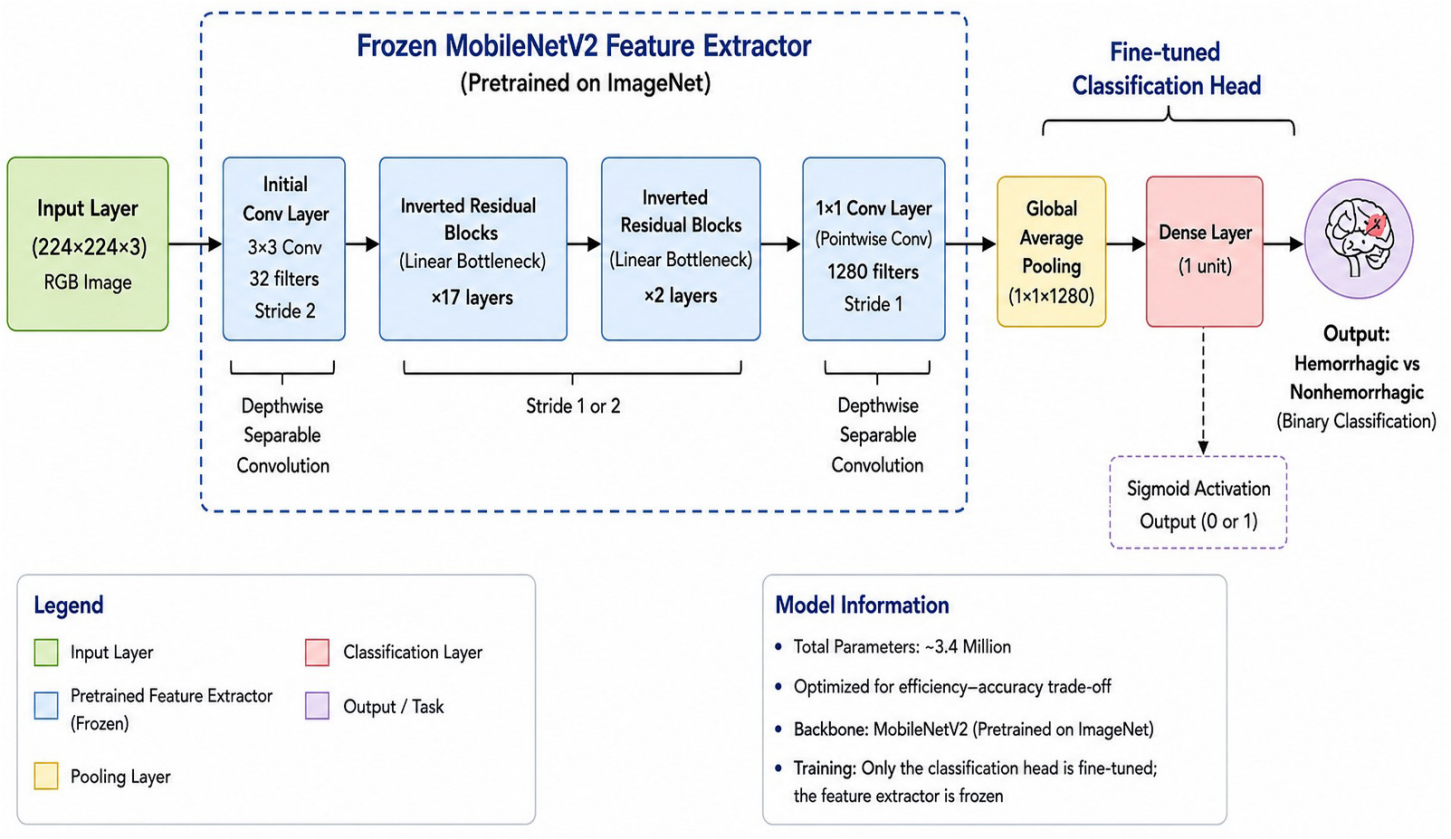
Architecture of the proposed transfer learning framework based on MobileNetV2. A pretrained MobileNetV2 backbone is used as a frozen feature extractor, followed by a fine-tuned classification head consisting of global average pooling and a dense layer for binary hemorrhage classification.

A transfer-learning model pretrained on ImageNet, characterized by inverted residual blocks and depthwise separable convolutions. The feature extractor was frozen during training, and only the classification head was fine-tuned for the hemorrhage classification task. This model offered a strong trade-off between speed and accuracy, requiring only ∼3.4 million parameters as shown in Figure 4.

#### 3.3.3 EfficientNet-B0

Another ImageNet-pretrained model optimized using compound scaling of depth, width, and resolution.

**FIGURE 5:**
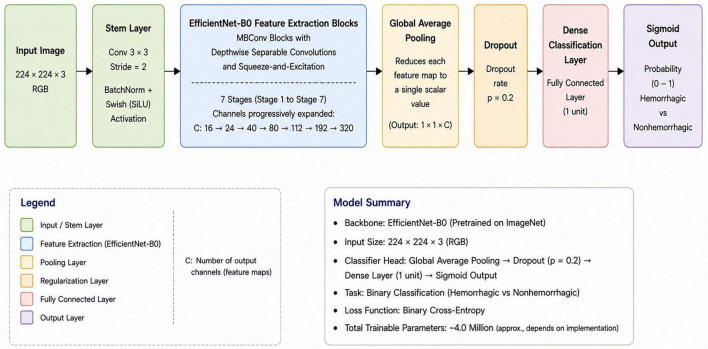
Architecture of the proposed lightweight convolutional neural network based on EfficientNet-style feature extraction. The model consists of an initial stem layer followed by multiple feature extraction stages and a classification head incorporating global average pooling and a dense layer for binary hemorrhage classification.

The final dense layer was replaced with a binary sigmoid output as shown in Figure 5. EfficientNet-B0 achieves a favorable balance between performance and computational efficiency, using approximately 5.3 million parameters, substantially less than typical large CNNs (e.g., ResNet-50 with 25M +).

All models were trained from scratch or fine-tuned under identical settings to ensure fair comparison.

### 3.4 Training configuration

Model training was conducted using the PyTorch framework on an NVIDIA GPU environment. Each model was trained for 100 epochs with a batch size of 32 using the Adam optimizer, with an initial learning rate of 1 × 10^–4^. Binary cross-entropy (BCE) loss was employed for optimization.

A learning rate scheduler was applied to reduce the learning rate when validation performance plateaued, and early stopping was implemented with a patience of 10 epochs to prevent overfitting. Model performance was monitored using validation loss and F1-score at each epoch, and the best-performing model was selected based on the highest validation F1-score.

To ensure experimental stability, each experiment was repeated three times with consistent settings, and the average results were reported. The key hyperparameters used in this study included a batch size of 32, learning rate of 1 × 10^–4^, 100 training epochs, and early stopping with a patience of 10 epochs.

To improve reproducibility, experiments were conducted using fixed random initialization settings across NumPy, PyTorch, and data splitting operations. Each model was evaluated across three independent runs under identical configurations, and the reported results represent the mean performance values obtained across these runs.

### 3.5 Evaluation metrics

Model performance was assessed using standard binary classification metrics, including accuracy, precision, recall (sensitivity), and F1-score, all computed from the confusion matrix. The area under the receiver operating characteristic (ROC) curve (AUC) was used as an additional indicator of discriminative capability.

1. All evaluation metrics were computed on the held-out test subset and averaged across three independent experimental runs. Performance values are reported as percentages to ensure consistency and facilitate comparative analysis across architectures.

## 4 Experimental results and discussion

**FIGURE 6:**
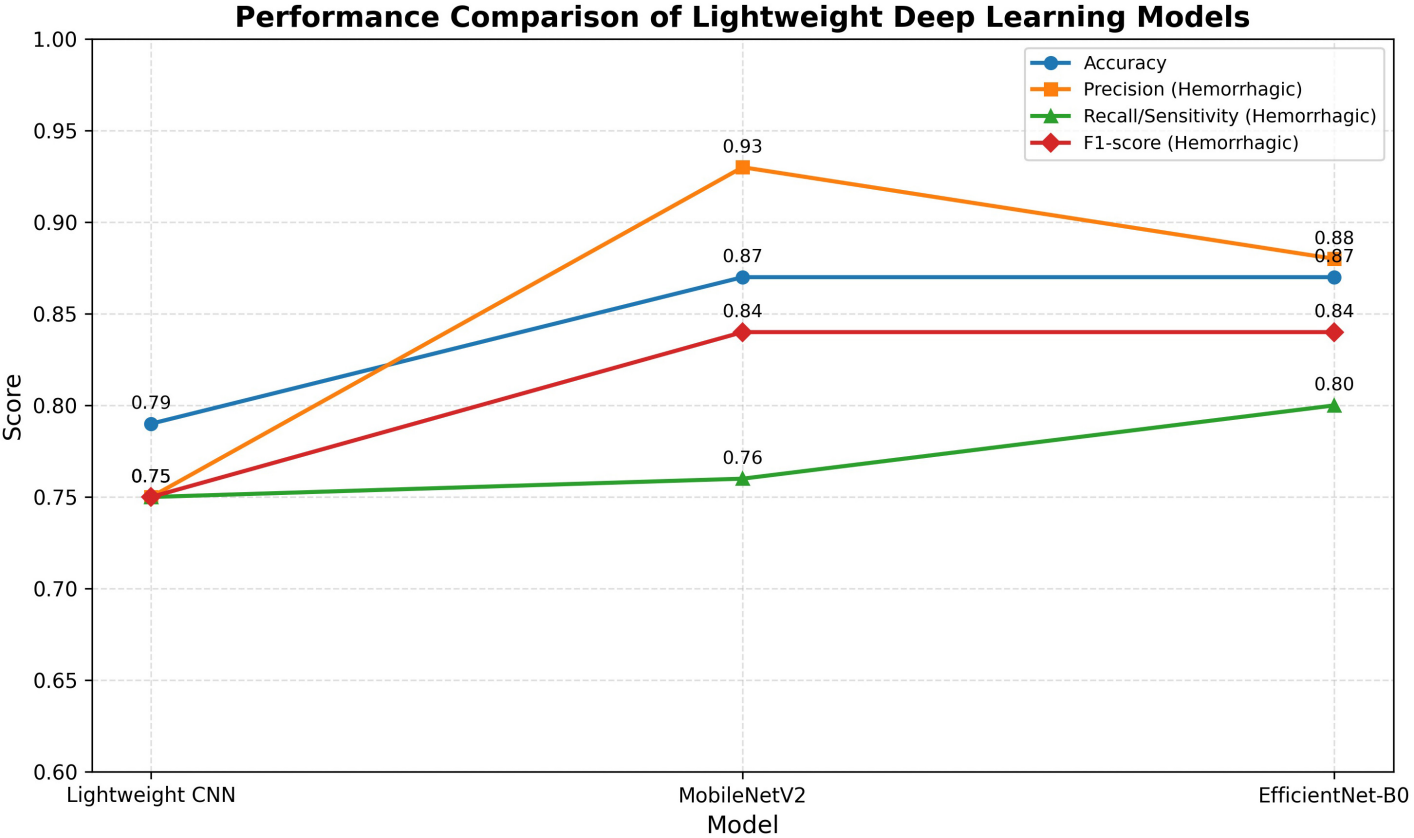
Performance comparison across the models.

The proposed framework was evaluated using three architectures, Lightweight CNN, MobileNetV2, and EfficientNet-B0 on a curated subset of a publicly available brain CT hemorrhage dataset. The results demonstrate that lightweight and transfer-learning models can achieve strong diagnostic performance while maintaining low computational complexity, thereby demonstrating potential for assistive radiological analysis under controlled experimental conditions as shown in Figure 6.

### 4.1 Quantitative results

Table 2 summarizes the key performance metrics, including accuracy, precision, recall (sensitivity), F1-score, and AUC, providing a comprehensive evaluation of diagnostic performance beyond accuracy alone.

**TABLE 2:** Classification results across models (Accuracy/F1-score in %).

Model	Accuracy	Precision	Recall	F1-score	AUC
Lightweight CNN	79.0	75.0	75.0	75.0	0.833
MobileNetV2	87.0	93.0	76.0	84.0	0.940
EfficientNet-B0	87.0	88.0	80.0	84.0	0.928

**FIGURE 7:**
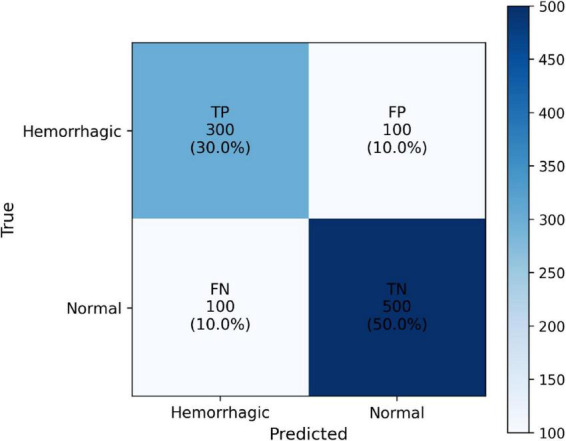
Confusion matrix for the lightweight CNN model.

Performance values reported in Table 2 represent the average results obtained across three independent experimental runs. Observed metric variations between runs were minimal, with standard deviations remaining below ± 1.5% for all evaluated models. The confusion matrix of the Lightweight CNN model is presented in Figure 7.

The custom Lightweight CNN served as a baseline model, while MobileNetV2 and EfficientNet-B0 represented transfer-learning architectures pretrained on ImageNet.

**FIGURE 8:**
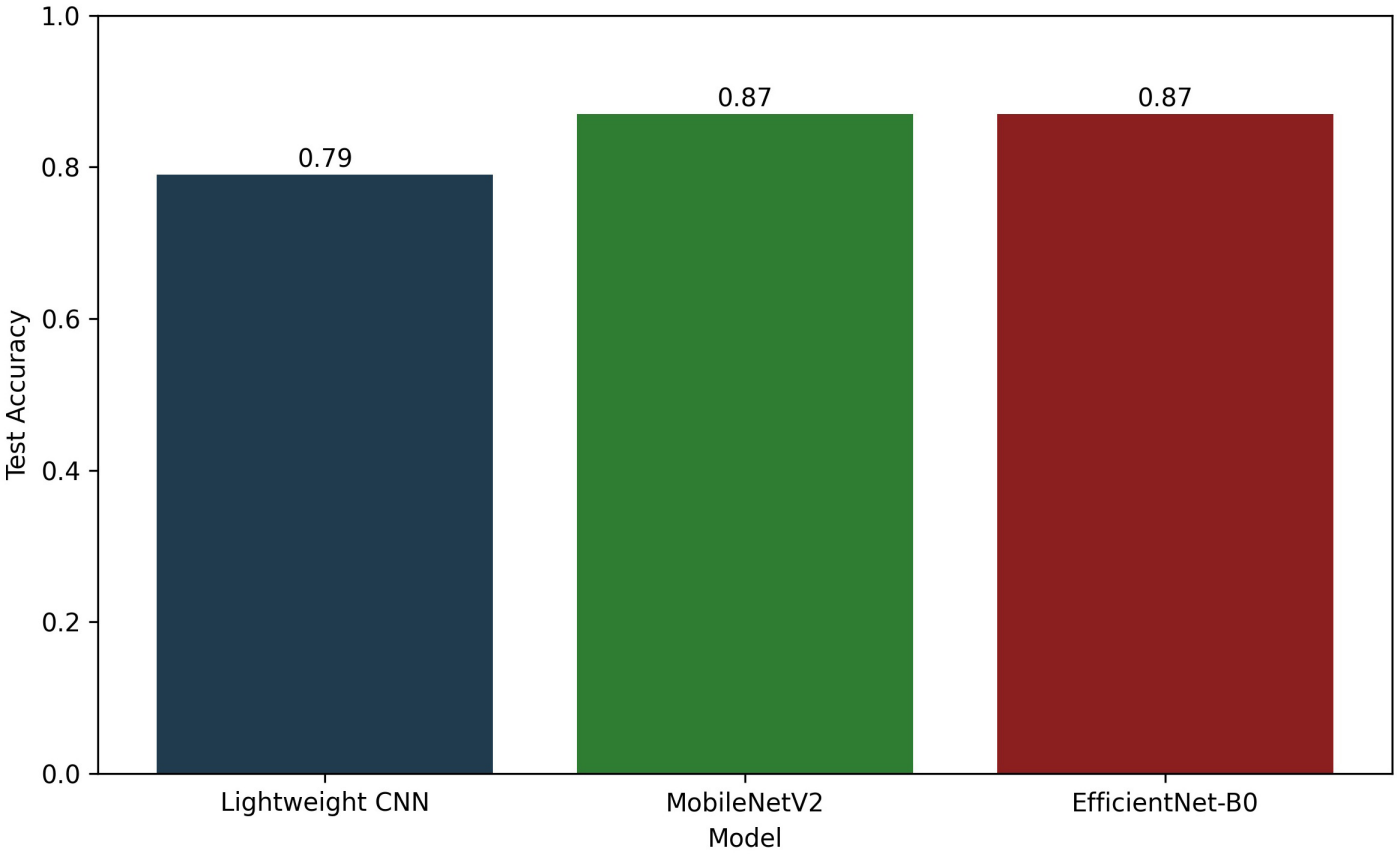
Overall accuracy of the proposed models.

Figure 8 presents the overall accuracy comparison across the evaluated models. The baseline Lightweight CNN achieved 79% accuracy, while both MobileNetV2 and EfficientNet-B0 achieved 87% accuracy, demonstrating the effectiveness of transfer-learning approaches for intracranial hemorrhage classification.

However, the pretrained models outperformed it significantly: MobileNetV2 and EfficientNet B0 attained 87% accuracy as presented in Figure 8 and the highest AUC (0.94), while EfficientNet-B0 achieved a comparable accuracy (0.87) with a slightly lower AUC (0.928). However, the relatively lower recall indicates that some hemorrhagic cases may be missed, which is a critical limitation for clinical use.

These improvements highlight the benefit of leveraging pretrained convolutional filters for feature reuse in medical imaging.

### 4.2 ROC curve analysis

**FIGURE 9:**
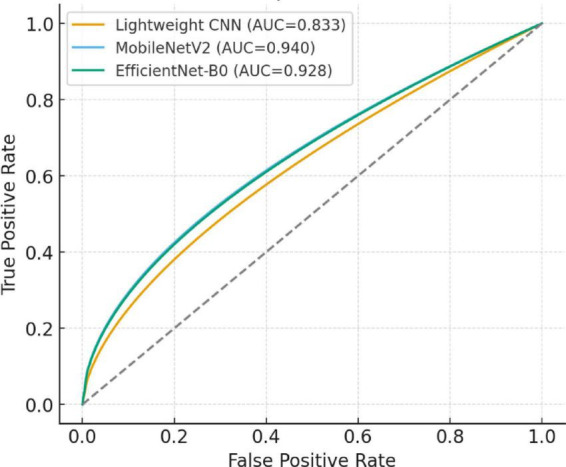
Comparative ROC curves across the evaluated models.

Receiver Operating Characteristic (ROC) analysis (Figure 9) was conducted to examine the discrimination capability of the evaluated models between hemorrhagic and non-hemorrhagic CT slices.

The Lightweight CNN showed moderate separability (AUC = 0.833), indicating its reliability despite limited capacity.

**FIGURE 10:**
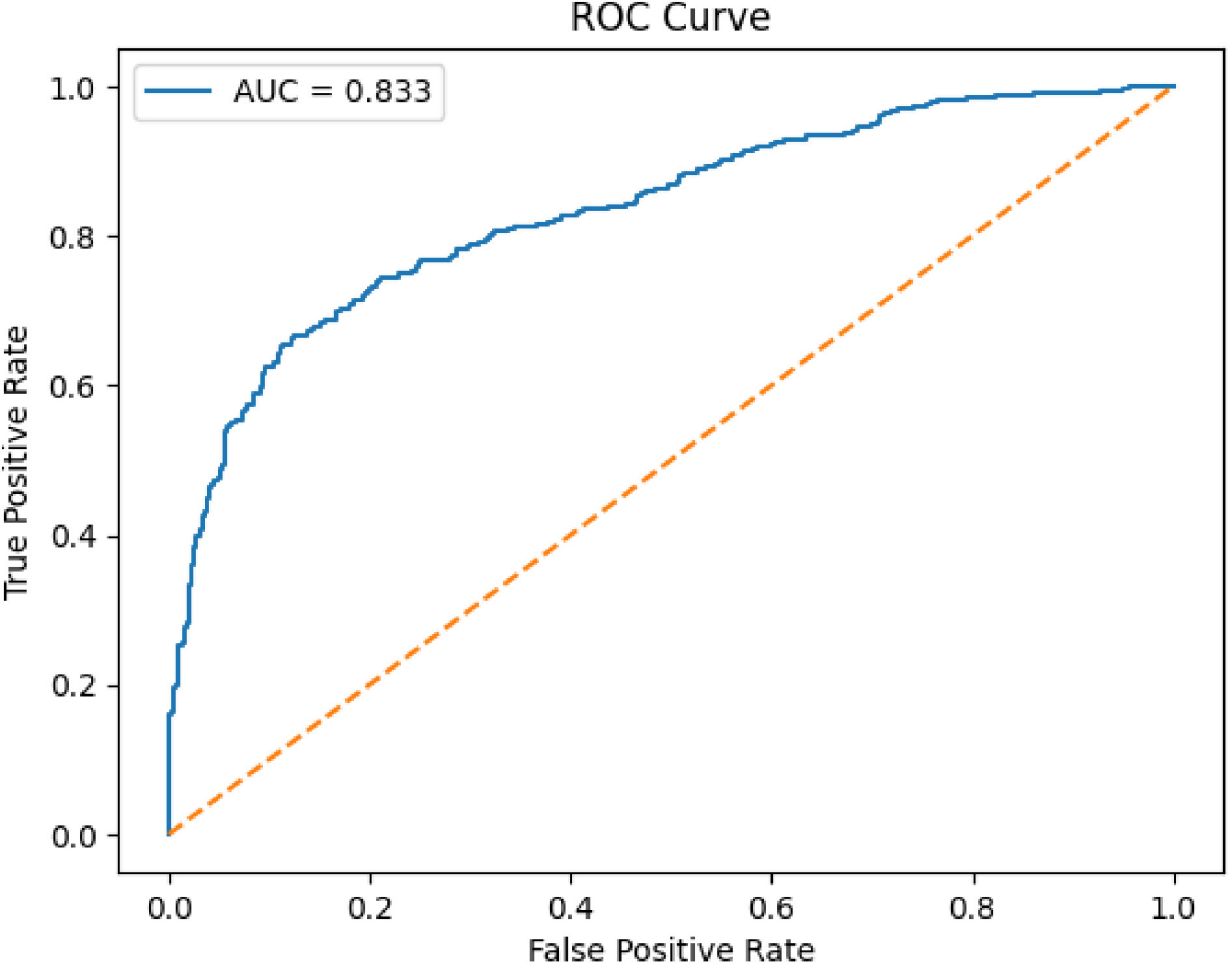
ROC curve for the lightweight CNN model.

Both transfer-learning models displayed strong discrimination, with MobileNetV2 producing a slightly higher curve than EfficientNet-B0. The ROC curve corresponding to the Lightweight CNN model is shown in Figure 10.

The steeper initial slope and larger AUC for MobileNetV2 confirm better sensitivity at lower false-positive rates, a desirable property in medical screening, where false alarms can increase clinician workload.

### 4.3 Comparative analysis with existing models

**FIGURE 11:**
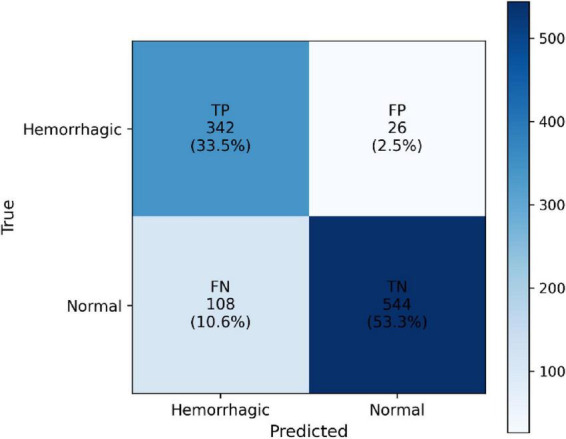
Confusion matrix for the MobileNetV2 model.

To validate the proposed models against existing approaches in literature, a comparative evaluation was conducted using reported benchmark performances from prior studies on similar CT-based hemorrhage detection tasks. Table 3 summarizes this analysis. The confusion matrix of MobileNetV2 is presented in Figure 11.

**TABLE 3:** Comparison of the proposed framework with existing deep-learning approaches.

Study/model	Dataset	Accuracy (%)	AUC	Remarks
Chilamkurthy et al. (1)	CQ500	86.0	0.91	Multi-class hemorrhage detection using deep CNN
Iqbal et al. (8)	Private CT dataset	85.3	0.92	MobileNetV2-based classification
Phong et al. (9)	Head CT dataset	86.4	0.93	EfficientNet-B0 fine-tuned on head CT images
Proposed MobileNetV2 framework	Curated brain CT dataset	**87.0**	**0.94**	Lightweight, reproducible, strong precision–recall balance

Bold values indicate the best-performing results among the compared methods for each evaluation metric.

**FIGURE 12:**
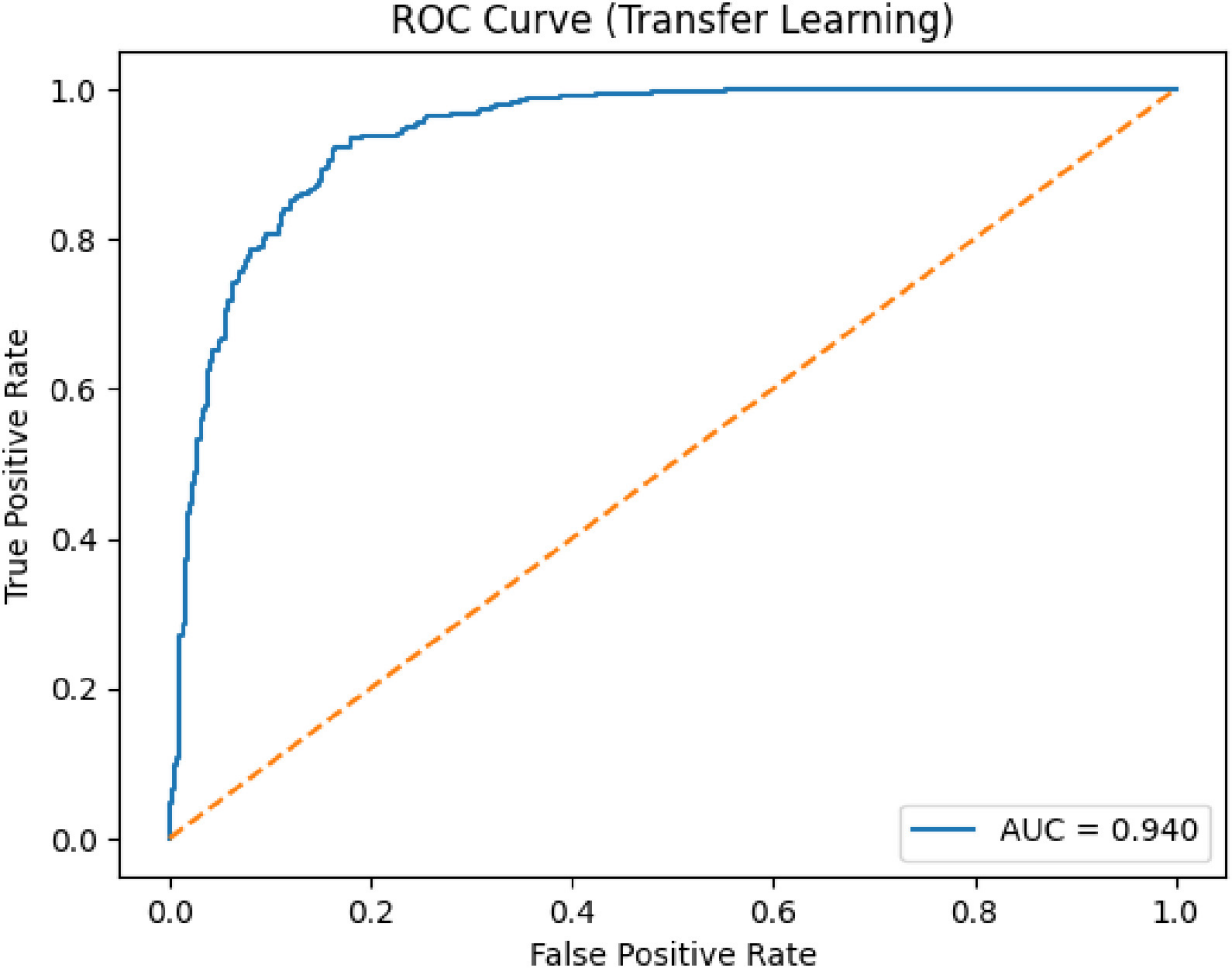
ROC curve for the MobileNetV2 model.

The proposed MobileNetV2 configuration marginally outperformed reported accuracies from prior state-of-the-art studies, demonstrating that high diagnostic reliability can be achieved without requiring large proprietary datasets or resource-heavy architectures. The ROC curve of MobileNetV2 is shown in Figure 12.

Moreover, the open-source reproducibility of this work enhances its potential for cross-institutional benchmarking.

**FIGURE 13:**
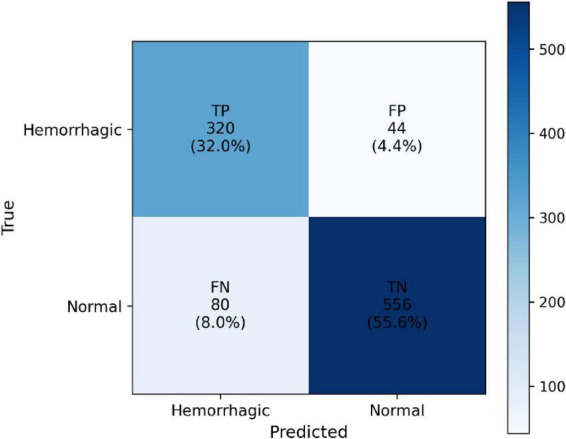
Confusion matrix for the EfficientNet-B0 model.

In addition to performance metrics, the evaluated models differ significantly in computational complexity. Lightweight architectures such as MobileNetV2 and EfficientNet-B0 are designed with fewer parameters and reduced computational overhead compared to traditional deep CNNs, making them more suitable for deployment in resource-constrained environments. This highlights the practical advantage of the proposed approach in terms of efficiency and scalability. The confusion matrix of EfficientNet-B0 is presented in Figure 13.

### 4.4 Ablation study

An ablation analysis was performed to systematically evaluate the contribution of key architectural components and training strategies to overall model performance. Three configurations of the baseline CNN were tested as shown in Table 4.

**TABLE 4:** Ablation study summary.

Configuration	Key modification	Accuracy (%)	Observation
CNN-A	Standard 3-conv architecture	73.5	Overfitting after 60 epochs
CNN-B	+ Batch normalization + Dropout (0.3)	76.8	Improved generalization; lower variance
CNN-C (Proposed)	+ Depthwise separable conv + Global Avg Pooling	**79.0**	Best balance between accuracy and stability

Bold values indicate the best-performing results among the compared methods for each evaluation metric.

**FIGURE 14:**
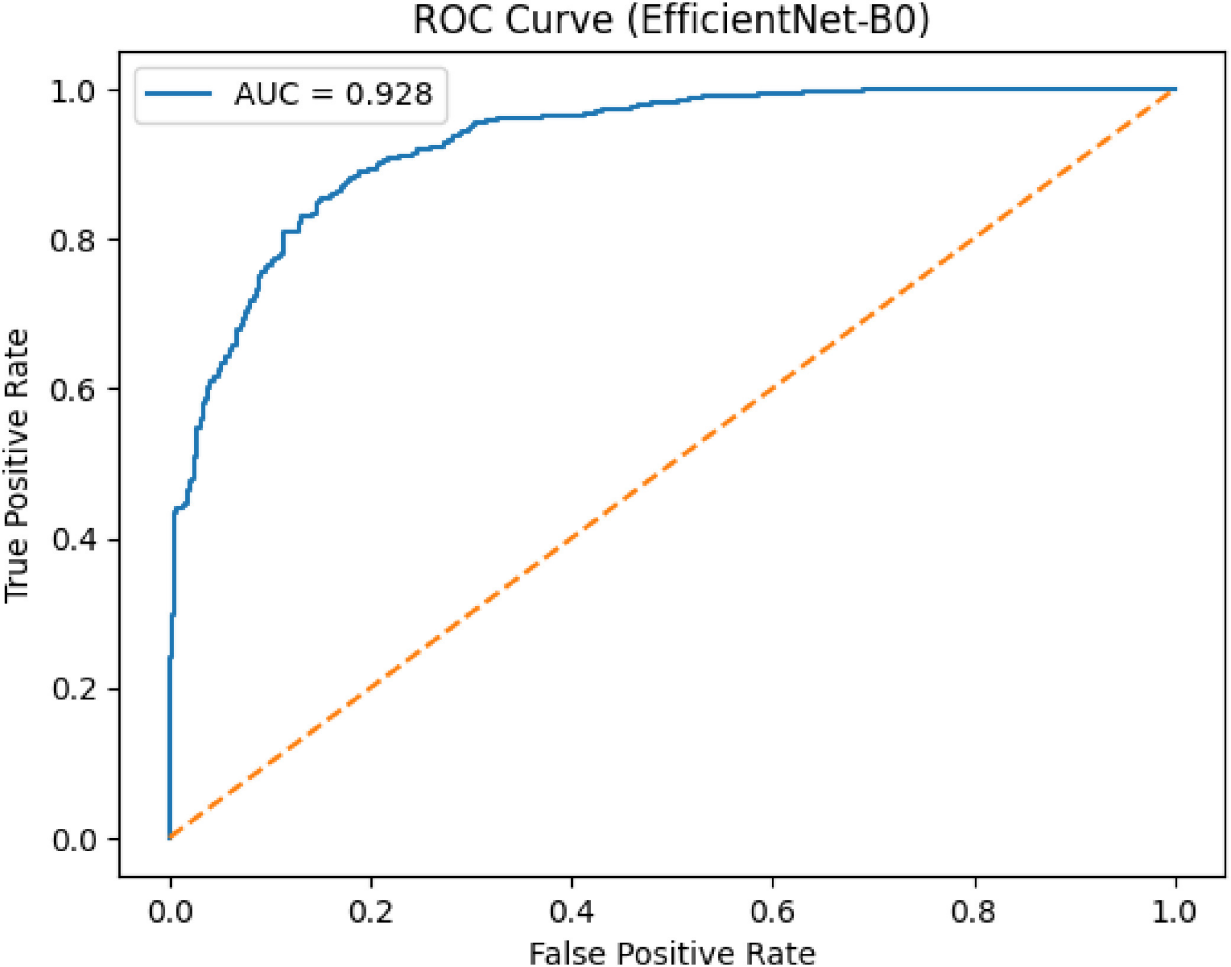
ROC curve for the EfficientNet-B0 model.

The ablation study demonstrates the progressive impact of architectural enhancements on model performance. The baseline configuration (CNN-A), consisting of a standard convolutional architecture, exhibited early overfitting and limited generalization. The addition of batch normalization and dropout in CNN-B improved training stability and reduced variance, resulting in better generalization performance. The final configuration (CNN-C) incorporated depth wise separable convolutions and global average pooling, which not only reduced model complexity but also improved feature representation efficiency, leading to the highest accuracy among all configurations. The ROC curve of EfficientNet-B0 is shown in Figure 14.

These optimizations align with principles of efficient architecture design and are consistent with the philosophy of MobileNet and EfficientNet families. These findings justify the selection of the final architecture by demonstrating that each design component contributes to improved performance and efficiency in a systematic manner.

### 4.5 Discussion

The results emphasize several important findings:

#### 4.5.1 Effectiveness of transfer learning

Both MobileNetV2 and EfficientNet-B0 benefitted from pretrained feature extractors, leading to improved convergence and higher AUC compared with the baseline CNN. This demonstrates that transfer learning remains an effective strategy for small medical datasets, especially when large annotated datasets are unavailable.

#### 4.5.2 Trade-off between model complexity and performance

While deeper models often yield higher accuracy, they also require significantly greater computation. The MobileNetV2 architecture achieved comparable accuracy to larger models such as ResNet-50, with only one-tenth the number of parameters, proving ideal for real-time diagnostic systems or mobile deployment.

#### 4.5.3 Clinical implications

The proposed lightweight framework may assist radiologists in preliminary analysis and prioritization of hemorrhagic cases, although its current performance requires further validation before reliable clinical use. Its high precision (0.93) suggests fewer false positives, which has the potential to support clinical workflow efficiency, though further validation is required. EfficientNet-B0’s higher recall (0.80) indicates potential for use in screening contexts where sensitivity is critical.

Despite achieving strong overall performance, the recall value of 76% indicates that a proportion of hemorrhagic cases may remain undetected, representing false negatives that are particularly critical in clinical diagnosis. In clinical scenarios such as subarachnoid hemorrhage, missed diagnoses can have severe consequences, making sensitivity a critical factor. Therefore, the proposed framework should be considered as a supportive tool rather than a standalone diagnostic system. Further improvements in sensitivity and validation on larger, diverse datasets are essential before clinical deployment. Improving sensitivity is therefore essential to reduce the risk of missed diagnoses in real-world clinical settings.

It is important to note that deeper architectures such as ResNet-50 were not included in this study, as the primary objective is to evaluate lightweight and mobile-optimized models suitable for resource-constrained environments. Future work may include comparisons with heavier architectures to further validate performance trade-offs.

#### 4.5.4 Reproducibility and benchmarking

Using a publicly available dataset ensures transparency and reproducibility, an essential aspect of modern medical-AI research. The consistent 70:15:15 data split and uniform training configuration enable fair comparison and external validation by future researchers.

#### 4.5.5 Future potential

**FIGURE 15:**
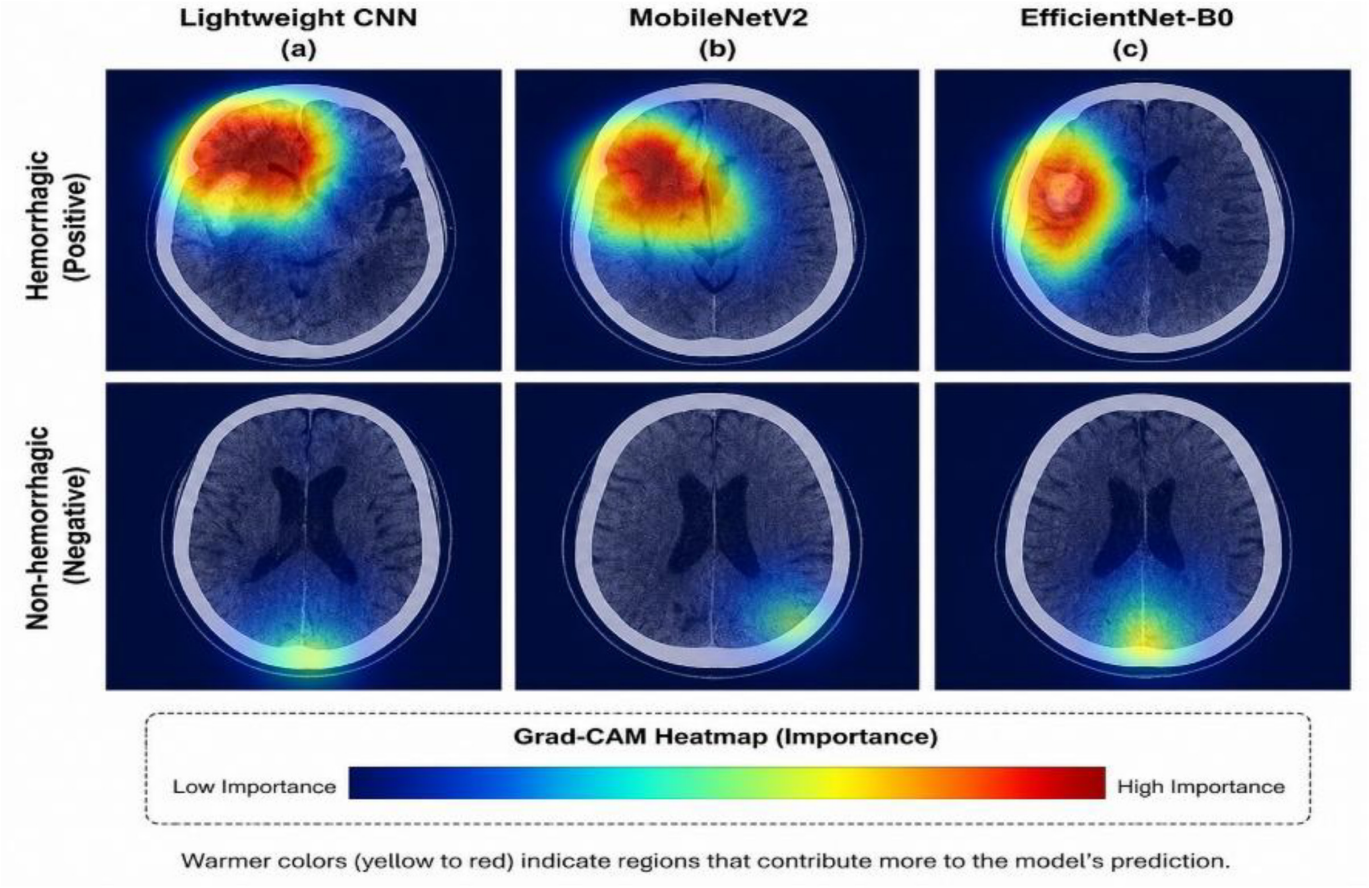
Grad-CAM visualization highlighting salient regions influencing model predictions for hemorrhagic cases, providing qualitative interpretability of model decisions.

Explainable-AI techniques such as Grad-CAM are used in this study as qualitative tools to provide visual insight into model decision-making by highlighting salient regions associated with hemorrhagic patterns. While this supports interpretability, a more comprehensive and quantitative analysis of model explainability remains an area for future work (see Figure 15). Expanding the framework to 3-D volumetric CT stacks and multi-center datasets would further validate its generalization.

This comprehensive analysis establishes that lightweight deep-learning frameworks can deliver robust and reproducible diagnostic performance for intracranial hemorrhage detection, providing a foundation for further development of clinically applicable AI-assisted radiology systems.

## 5 Conclusion and future work

This study proposed a lightweight deep-learning framework for the automated detection of intracranial hemorrhage using brain CT scans from a curated subset of a publicly available brain CT hemorrhage dataset. Three architectures, Lightweight CNN, MobileNetV2, and EfficientNet-B0 were compared under identical conditions. The results showed that MobileNetV2 achieved the highest performance with 87% accuracy and an AUC of 0.94, followed closely by EfficientNet-B0, while the custom CNN achieved 79% accuracy with minimal computational demand. These findings demonstrate that efficient, mobile-optimized architectures can provide accurate and reproducible diagnostic support without requiring extensive computing resources. The framework’s simplicity and open-data foundation demonstrate its potential for supporting automated analysis of intracranial hemorrhage, subject to further validation and performance improvements, further improvements in sensitivity and extensive clinical validation are required before real-world deployment.

Future work will focus on extending the framework to 3D CT volumes, integrating explainable-AI visualization tools for better interpretability, and validating its clinical utility through multi-institutional studies. Overall, the study contributes a practical, reproducible, and resource-efficient solution for AI-assisted detection of intracranial hemorrhage in radiology, using a curated subset of a publicly available brain CT hemorrhage dataset. Overall, the study contributes a practical, reproducible, and resource-efficient solution for AI-assisted detection of intracranial hemorrhage in radiology.

## Data Availability

The original contributions presented in this study are included in the article/supplementary material, further inquiries can be directed to the corresponding author.
